# PARP inhibitors as maintenance therapy in ovarian cancer after platinum-sensitive recurrence: real-world experience from the Unicancer network

**DOI:** 10.1093/oncolo/oyaf075

**Published:** 2025-05-11

**Authors:** Nicolas Rippstein, Christophe Zemmour, Manuel Rodrigues, Isabelle Ray-Coquard, Laurence Gladieff, Patricia Pautier, Jean-Sébastien Frénel, Hélène Costaz, Coriolan Lebreton, Christophe Pomel, Pierre-Emmanuel Colombo, Frédéric Marchal, Cécile Guillemet, Thibault de la Motte Rouge, Lauriane Eberst, Lise Bosquet, Elise Deluche, Renaud Sabatier

**Affiliations:** Medical Oncology Department, Limoges University Hospital, Limoges, France; Department of Clinical Research and Investigation. Biostatistics unit. Paoli-Calmettes Institute, Marseille, France; Medical Oncology Department, Curie Institute, University of Paris, Paris, France; Medical Oncology Department, Léon Bérard Cancer Centre, Lyon, France; Medical Oncology Department, Oncopole Claudius Régaud IUCT, Toulouse, France; Medical Oncology Department, Gustave Roussy, Villejuif, France; Institut de Cancérologie de L’Ouest, Medical Oncology Department, Saint-Herblain, France; Surgical Oncology Department, Georges-François Leclerc Cancer Center, Dijon, France; Medicla Oncology Department, Institut Bergonié, 12 ARTiST lab, Inserm U1312, Université de Bordeaux, Bordeaux, France; Surgical Oncology Department, Jean Perrin Cancer Center, Unité Inserm IMOST, Université de Clermont Auvergne, Clermont-Ferrand, France; Surgical Oncology Department, Montpellier Cancer Institute, Montpellier, France; Surgical Oncology Department, Lorraine Cancer Institute, Université de Lorraine, Nancy, France; Medical Oncology Department, Henri Becquerel Cancer Center, Rouen, France; Medical Oncology Department, Eugène Marquis Cancer Center, Rennes, France; Medical Oncology Department, Institut de Cancérologie de Strasbourg Europe, Strasbourg, France; Health Data and Partnerships Departement, Unicancer, Paris, France; Medical Oncology Department, Limoges University Hospital, Limoges, France; Aix-Marseille Univ, CRCM, Inserm, CNRS, Institut Paoli-Calmettes, Department of Medical Oncology, Marseille, France

**Keywords:** real word data, PARP inhibitor, platinum-sensitive, maintenance therapy, recurrent ovarian cancer

## Abstract

**Background:**

Based on results of randomized clinical trials, polyADP‐ribose polymerase inhibitors (PARPi) have become the standard of care in patients with platinum-sensitive recurrent ovarian cancer (OvC) in patients responding to platinum chemotherapy. However, little is known about their impact on survival in a real-world setting.

**Patients and methods:**

This retrospective French multicenter observational study included women with platinum-sensitive recurrent OvC (not limited to the first platinum-sensitive relapse) receiving PARPi as maintenance after response to platinum-based chemotherapy. They were compared to patients with similar characteristics undergoing observation after chemotherapy completion. Data were collected in the Ovarian Cancer Epidemiological Strategy and Medical Economics (ESME-OC) database between 2011 and 2021. We explored progression-free survival (PFS) and overall survival (OS) benefits with PARPi maintenance.

**Results:**

One hundred and twenty-three patients matching the selection criteria were included in the PARPi group and 397 patients in the control group. Median PFS was 19.9 months (95CI [15.0-21.9]) in the PARPi group vs 13.4 months (95CI [11.8-15.0]) in the control group, with a HR = 0.71 (95CI [0.55-0.93]), *P* = .01). Median OS was 82.0 months (95CI [48.6-Not Estimable]) in the PARPi group *vs* 44.7 months (95CI [38.8-53.7]) in the control group (HR = 0.47, 95CI [0.30-0.74], *P* < .001). Multivariate analyses including performance status, histological subtype, achievement of cytoreductive surgery at relapse, and platinum-free interval, confirmed the independent prognostic impact of PARPi treatment.

**Conclusion:**

This first national study focusing on the efficacy of PARPi in a real-world population shows similar benefits than in randomized clinical trials, supporting their use in clinical routine practice.

**Database registration:**

clinicaltrials.gov Identifier NCT03275298.

**Implications for practice**

In this real-world study on PARPi maintenance after platinum-sensitive relapse in ovarian cancer, PARPi group showed improved PFS (19.9 months) vs control group (13.4 months, HR = 0.71, 95CI [0.55-0.93], *P* = .01).

Risk of death was decreased in PARPi group (OS HR = 0.47, 95CI [0.30-0.74], *P* < 0.001) but needs to be confirmed with longer follow-up.

This real-world study confirms results of randomized phase 3 trials in a non-selected population, supporting their use in clinical routine practice.

## Background

Ovarian cancer (OvC) ranks as the eighth most common cancer among women and represents the most lethal gynecological cancer in western countries.^1^ In France, there are approximately 5200 new cases diagnosed each year, resulting in over 3400 deaths.^2^ Curative treatment options are limited to the initial phase of the disease. Unfortunately, more than 70% of patients diagnosed with advanced-stage ovarian cancer (FIGO stage III-IV) experience a relapse following standard first-line therapies. Recurrences occurring more than six months after platinum-based therapy completion are classified as platinum-sensitive relapses, whereas those occurring during chemotherapy or within the first six months after the completion of first-line treatment are referred to as platinum-refractory/resistant, making patients ineligible for platinum rechallenge.^3^

The current systemic standard therapy for platinum-sensitive recurrent OvC is a platinum-based doublet.^4^ Patients who have not have received PARP (poly (ADP‐ribose) polymerase) inhibitors (PARPi) maintenance after first-line platinum-based chemotherapy become candidates to PARPi maintenance after second-line chemotherapy in case of response (complete or partial response) to platinum-based chemotherapy.^4^ PARPi induce a trapping of PARP on DNA at sites of single-strand breaks, resulting in the collapse of the replication fork and cell death.^5^ In cells with homologous recombination deficiency, including but not limited to *BRCA1/2* mutations, this mechanism results in synthetic lethality.^6^ Olaparib, niraparib, and rucaparib are the three PARPi approved for use in the platinum-sensitive recurrent setting.^7-11^ In patients with *BRCAm (BRCA1/2* mutated) tumors enrolled in the SOLO-2 trial, olaparib maintenance after platinum-based chemotherapy significantly increased progression-free survival (PFS) with a median of 19.1 months compared to 5.5 with placebo.^7^ In non-BRCA selected patients, both niraparib and rucaparib improved PFS compared to placebo, with hazard ratios (HR) for PFS ranging from 0.36 to 0.45. Consequently, these drugs received regulatory approval regardless of BRCA status.^8,9^

Randomized pivotal trials were not adequately powered to assess overall survival (OS), and the final OS analyses reported conflicting results. Olaparib tended to improve OS in relapsing *BRCA*m tumors (HR = 0.74, 95CI [0.54-1]).^10^ No benefit was observed with niraparib, either in the *BRCA*m (HR = 0.85, 95CI [0.61-1.20]) and *BRCA* wild-type populations (HR = 1.06, 95CI [0.81-1.37]).^12^ Similar findings were noted with rucaparib in both the *BRC*Am (HR = 0.83, 95CI [0.58-1.19]) and the *BRCA*-unselected populations (HR = 0.99, 95CI [0.81-1.22]).^13^ Furthermore, apart from the uncertain long-term efficacy, PARPi use has been associated with higher rates of hypertension and hematological toxicity, including rare but fatal myelodysplastic syndromes and acute myeloid leukemia.^7,13,14^

Consequently, there is a critical need for real-world data to comprehensively investigate the efficacy and safety profiles of PARPi in the recurrent ovarian cancer setting, as large prospective trials may not adequately represent the routine oncology population, potentially introducing selection bias by including only well-fit patients.

While prospective phase IV trials serve as the gold standard for evaluating treatments in real-world settings, they primarily focus on safety-related observations and may not be suitable to address questions involving multiple drugs. This study, conducted on behalf of the Unicancer network, is the first national investigation to focus on PARPi efficacy in a real-world population, assessing both PFS and OS in this clinical context.

## Materials and methods

### ESME-OC database and cohort selection

The UNICANCER Epidemiological Strategy and Medical Economics Ovarian Cancer (ESME-OC) database (NCT03275298) is a real-world retrospective French multicenter prospectively maintained database that collects exhaustive data of all consecutive patients, treated for ovarian cancer from 2011 to 2021 in one of the 18 French Comprehensive Cancer Centers of the Unicancer network. This database compiles data from Patient’s Electronic medical records (EMR), inpatient hospitalization records, and pharmacy records.^15^

Patients included in this analytical study were recruited between January 01, 2011 and December 31, 2019. Data were collected until the cut-off date (January 3, 2022), date of death, or date of last contact in the center if lost to follow-up. Patients’ demographics, cancer characteristics, pathology, outcomes, and treatments were collected.

Selection criteria were the following: female ≥ 18 years old, recurrent adenocarcinoma of the ovary, fallopian tubes, or peritoneum (carcinosarcoma were also allowed), time from prior platinum-based chemotherapy ≥ 6 months, objective response to the last platinum-based regimen administered in the recurrent setting, treatment with PARP inhibitor (olaparib, niraparib, or rucaparib) as maintenance after platinum-based chemotherapy, not limited to the first platinum-sensitive relapse. The non-selection criteria were concomitant use of bevacizumab, immune checkpoint inhibitor, placebo within randomized clinical trials, or targeted therapies other than PARP inhibitors in the recurrent setting.

Inclusion criteria were similar for the control group, except for the use of PARPi for recurrent disease, whatever the line. They were included before PARPi approvals and did not receive PARPi because of contraindications or patient/physician’s choice. To avoid the inclusion of patients without response after platinum-based chemotherapy, patients with time from chemotherapy completion to subsequent chemotherapy shorter than three months were excluded from the control group. Due to a lack of data related to response to chemotherapy in late lines, inclusion in the control group was limited to the first platinum-sensitive relapse.

### Objectives of the study

The primary objectives were to assess PFS and OS in patients receiving PARPi as maintenance therapy after platinum-sensitive relapse. PFS and OS were compared between patients treated with PARPi and those who did not receive maintenance therapy in the recurrent setting.

Secondary objectives were to evaluate the clinical, pathological, and molecular characteristics correlated to efficacy, and to explore the causes of PARPi discontinuation in this real-world cohort.

### Data collection and statistical analysis

The level of statistical significance was set at *α* = 0.05. All tests were two-sided. Statistical analyses were performed with the SAS® 9.4 software. Categorical variables were described using counts and frequencies (calculated on the total of available data), and quantitative variables were described using medians and ranges. Patients’ characteristics of the PARPi arm and control arm were compared using Chi-square or exact Fisher tests for qualitative variables, and rank-Wilcoxon tests for quantitative variables.

PFS was defined as the time from the first chemotherapy cycle in the recurrent setting to disease progression (local assessment based on clinical, radiological, and biological criteria) or death. OS was defined as the time from the first chemotherapy cycle in the recurrent setting to death. Patients without considered events were right-censored at the date of their last follow-up.

Univariate and multivariate analyses of PFS and OS were performed to assess the prognosis impact of the following criteria: treatment group (PARPi *vs* observation), performance status (0-1 vs 2, 0-1 vs 3-4), histological type (serous vs other), cytoreductive surgery for recurrent disease, time free interval between the last platinum chemotherapy lines (≤ 12 months vs > 12 months). PFS and OS arm effects were also assessed in a multivariate Cox regression including covariates significantly associated with survival in univariate analysis and with less than 30% of missing data. Hazard ratios were provided with their Wald bilateral confidence intervals and tests *P*-values for significance. Follow-up was estimated using the reverse Kaplan–Meier method. Patients lost to follow-up or without event were right censored at the date of their last follow-up. Survival curves were estimated using the Kaplan–Meier method, and the median PFS and OS were calculated with their bilateral confidence intervals.

To explore PARPi safety, and as more detailed data are not available in the ESME database, we collected AE leading to drug discontinuation, including hematological AEs and secondary hematological malignancies.

#### Ethical approval

The ESME-OC database was authorized by the French data protection authority (NCT03275298; initial authorization N°DE- 2017-311 and subsequent amendment obtained on October 14, 2019 in accordance with European regulations) and is managed by Unicancer in accordance with current best practice guidelines.^16^ No formal dedicated informed consent is required but patients are informed about the re-use of their electronically recorded data.

This work has been done according to Strengthening the Reporting of Observational Studies in Epidemiology (STROBE) criteria and the recently published ESMO guidelines for Reporting Oncology real-world evidence (GROW).^17,18^

## Results

### Patient baseline characteristics

**Figure 1.**
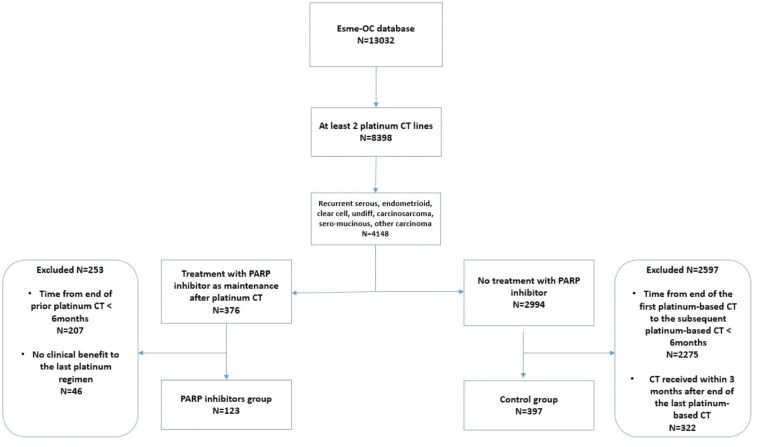
Flowchart of patients selected from the ESME-OC database. Abbreviations: CT, chemotherapy, ESME-OC, Epidemiological Strategy and Medical Economics Ovarian Cancer (ESME-OC).

Among the 13 032 included in the ESME-OC database, 8398 received at least 2 platinum chemotherapy lines and 4148 were recurrent serous, endometrioid, clear cell, undifferentiated, carcinosarcoma, sero-mucinous, or other carcinoma not otherwise specified. Among them, 123 patients matching the selection criteria were included in the PARPi group, whereas 397 patients were included in the control group (**Figure 1**).

The median age at diagnosis was 64 years (range 23-90), (Table 1). Most patients had a performance status of 0-1 (90%). Seventeen patients (14%) and 28 patients (8%) had a prior personal history of breast cancer in PARPi and control group, respectively (*P* = 0.04). Fifty patients (44%) in the PARPi group and 26 patients (12%) in the control group had a deleterious *BRCA1/2* mutation (*P* < 0.001). Other clinicopathological features were well balanced.

**Table 1.** Tumors and characteristics.

Parameter	Whole population (*n* = 520)	PARP inhibitors group (*n* = 123)	Control group (*n* = 397)	*P*-value
Age (years) at initial diagnosis				
Median [min-max]	64 [23- 90]	66 [42- 85]	63 [23- 90]	.16
<50 years old	59 (11.3)	11 (8.9)	48 (12.1)	.42
≥50 years old	461 (88.7)	112 (91.1)	349 (87.9)	
Performance status				
0-1	373 (89.9)	103 (93.6)	270 (88.5)	.35
2	32 (7.7)	6 (5.5)	26 (8.5)	
3-4	10 (2.4)	1 (0.9)	9 (3.0)	
Missing data	105	13	92	
Personal history of breast cancer				
No	445 (90.8)	103 (85.8)	342 (92.4)	.04
Yes	45 (9.2)	17 (14.2)	28 (7.6)	
Missing data	30	3	27	
Personal history of cancer associated with Lynch syndrome				
No	483 (98.6)	119 (99.2)	364 (98.4)	1
Yes	7 (1.4)	1 (0.8)	6 (1.2)	
Missing data	30	3	27	
Histological subtype				
Serous	393 (75.6)	113 (91.9)	280 (70.5)	<.001
Other	127 (24.4)	10 (8.1)	117 (29.5)	
*BRCA1/2* status				
*BRCA*mut	76 (22.8)	50 (43.9)	26 (11.8)	<.0001
Germline mutation	43 (56.6)	29 (58)	14 (53.8)	
Somatic mutation	19 (25)	13 (26)	6 (23.1)	
Not specified	14 (18.4)	8 (16)	6 (23.1)	
*BRCA* wild-type	258 (77.2)	64 (56.1)	194 (88.2)	
Missing data	186	9	177	

Data are *n* (%) unless otherwise specified.

Patients in the PARPi group were diagnosed in 2014, with most of the inclusions occurring in 2017. Patients in the control group received second-line platinum chemotherapy with little variation through the inclusion period (**Supplementary Figure 1**).

### Treatments received

The median number of cycles of platinum-based chemotherapy was 6 (range 1-34) in both groups. In the PARPi group, 52 patients (42%) received 2 lines of chemotherapy before maintenance treatment, 48 patients (39%) received 3 lines of chemotherapy before maintenance, and 23 patients (19%) received at least 4 lines of chemotherapy before maintenance PARPi treatment. As detailed in the inclusion criteria, all patients in the control group were in the first platinum-sensitive relapse setting.

Median treatment-free interval (TFI) between the 2 last platinum-based regimens was 29.9 months in the PARPi group vs. 26.9 months in the control group (*P* = 0.60). More than 80% of the patients had a TFI longer than 12 months in both groups (83.7% vs. 80.1%). Cytoreductive surgery at relapse was achieved in 20 patients (32.8% of cases with data available) in the PARPi group vs 47 patients (20.2% of patients with data available) in the control group.

Most patients included in the PARPi group received niraparib (52%) or olaparib (44%). Five patients (4%) received rucaparib. Median PARPi treatment duration was 75 days (range 3 – 2108). Twenty-six patients (21.1%) discontinued PARPi due to disease progression. Treatment discontinuation due to toxicity was reported in 53 (43.1%) patients. Most of them were related to hematological adverse events (28 patients, 22.8%) or digestive disorders (6.5%). Other causes were skin, renal, and cardiologic toxicities (1 patient each), and hepatitis (2 patients). Reasons for treatment discontinuation due to toxicities were not detailed in 12 (9.8%) patients. No secondary leukemia or myelodysplastic syndrome was reported in the PARPi population.

### Progression-free survival analysis

Median follow-up was 51.2 months (95CI [43.6-60.3]) in the whole population. It was longer in the control group (73.2 months, 95CI [65.6-80.0]) than in the PARPi subset (24.7 months, 95CI [20.5-29.3]).

**Figure 2.**
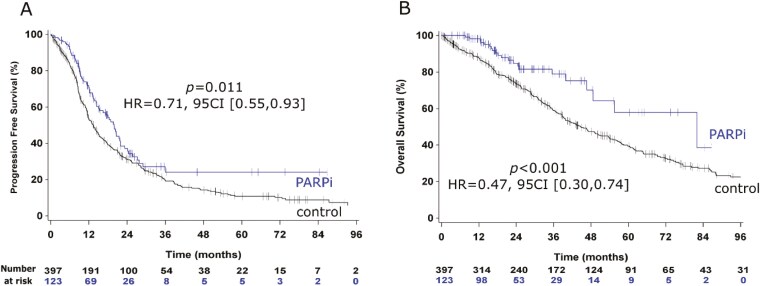
Kaplan-Meier curves for progression-free survival (A) and overall survival (B) in the control group (black curves) vs PARP inhibitor group (blue curves). *P* = Log-rank *P*-values.

Median PFS was 14.4 months (95CI [13.0-16.5]) for the whole population, (Supplementary Figure 2A). It was 19.9 months (95CI [15.0-21.9]) in the PARPi group *vs.* 13.4 months (95CI [11.8-15.0]) in the control group, with a HR = 0.71 (95CI [0.55-0.93], *P = *0.01), (Figure 2A). The 2y-PFS rates were 37% (95CI [27-47]) and 31% (95CI [26-36]) in PARPi and control groups, respectively.

In the whole population, PARPi maintenance, achievement of cytoreductive surgery at relapse, performance status, TFI ≤ 12 months, and histology were associated with better PFS (Table 2). Multivariate analysis showed that all tested parameters but the histological subtype remained significantly associated with PFS.

**Table 2.** Univariate and multivariate analysis for progression-free survival.

Variables	Classes	Univariate analysis			Multivariate analysis (*N* = 415)	
		*n*	HR [95%CI]	*P*-value	HR[95% CI]	*P*-value
**Performance status**	0-1	373				
	2	32	1.97 [1.30-2.97]	.001	1.62 [1.06-2.46]	.025
	3-4	10	2.01 [0.99-4.07]	.052	2.30 [1.13-4.68]	.021
**Histological subtype**	Other	127				
	Serous	393	0.80 [0.64-1]	.048	0.81 [0.62-1.04]	.102
**Treatment group**	Control	397				
	PARPi	123	0.71 [0.55-0.93]	.011	0.69 [0.51-0.92]	.011
**Cytoreductive surgery at relapse**	No	226				
	Yes	294	0.52 [0.43-0.64]	<.001	0.61 [0.48-0.78]	<.001
**TFI between the two last platinum-chemotherapy lines**	>12 months	421				
	≤12 months	99	2.52 [1.97-3.22]	<.001	2.25 [1.68-3.01]	<.001

Abbreviations: 95CI, 95% confidence interval; HR, hazard ratio; *N*, Number of patients; TFI, time-free interval..

As median follow-up was shorter in the PARPi subset than in the control group, we then explored PFS by censoring survival data at 24 months, corresponding to the median follow-up of the PARPi group. Benefits from PARPi were maintained with HR = 0.72 (95CI [0.55-0.95], *P *= .022).

**Figure 3.**
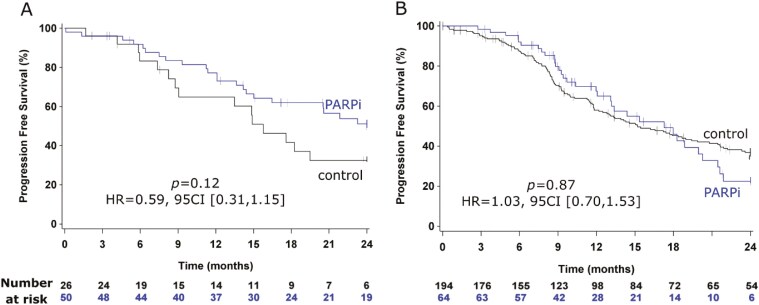
Kaplan-Meier curves for progression-free survival with censoring at 24 months. (A) BRCAmut patients; (B) BRCA wild-type patients. Control group (black curves) vs PARP inhibitor group (blue curves). *P* = Log-rank *P*-values. All regular figures (in order of citation within the manuscript text). All regular tables (in order of citation within the manuscript text).

We then performed an exploratory analysis of PARPi impact on PFS according to *BRCA*m status. In the *BRCA*m population, PFS tended to be improved with a median not reached after a 24-month follow-up in *BRCA*m tumors *vs.* 15.8 months in the control group (HR = 0.59, 95CI [0.31-1.15], *P* = .12) (Figure 3A). The same analysis in *BRCA* wild-type cases showed no benefit of PARPi vs. controls (median PFS of 17.3 months vs. 15.2 months in controls, HR = 1.03, 95CI [0.70-1.53], *P* = .87) (Figure 3B).

We then tried to explore the impact of a chemotherapy regimen on PARPi maintenance efficacy according to *BRCA* status.^19^ We observed no significant discrepancy regarding chemotherapy regimen according to *BRCA* status in our set: carboplatin/paclitaxel 34 in *BRCA*mut vs. 27% in *BRCA*wt; carboplatin/pegylated liposomal doxorubicin 43% vs. 48%; carboplatin/gemcitabine 7% vs. 20%; *P* = .11, Wilcoxon test. Due to the small subgroups sample size and limited number of events, we could not analyze survival with PARPi maintenance according to chemotherapy regimen and *BRCA* status. However, chemotherapy regimen distribution suggests that the differential PARPi effect we observed was not influenced by the chemotherapy regimen.

### Overall survival analysis

The median OS was 49.3 months (95CI [42.9-57.1]) in the whole population (Supplementary Figure 2B). Median OS was 82.0 months (95CI [48.6-NE]) and 44.7 months (95CI [38.8-53.7) in PARPi and control groups, respectively (HR = 0.47, 95CI [0.30-0.74], *P* < 0.001), (Figure 2B). Two-years OS rates were 85% (95CI [75-91]) and 74% (95CI [69-78]) in PARPi and control group, respectively. Other features correlated with OS were poor performance status (HR = 3.65, 95CI [1.79-7.43]), serous histology (HR = 0.77, 95CI [0.60-1.00]), cytoreductive surgery at relapse (HR = 0.49, 95CI [0.38-0.62]), and TFI ≤ 12 months (HR = 2.83, 95CI [2.13-3.74]), (**Table 3**). All variables remained significantly associated with OS in multivariate analysis.

**Table 3.** Univariate and multivariate analysis for overall survival

Variables	Classes	Univariate analysis			Multivariate analysis (*N* = 415)	
		*n*	HR [95%CI]	*P*-value	HR [95% CI]	*P*-value
**Performance status**	0-1	373				
	2	32	1.91 [1.17-3.10]	.009	1.56 [0.95-2.55]	.08
	3-4	10	3.65 [1.79-7.43]	<.001	4.68 [2.26-9.68]	<.001
**Histological subtype**	Other	127				
	Serous	393	0.77 [0.60-1]	.048	0.74 [0.56-0.99]	.045
**Treatment group**	Control	397				
	PARPi	123	0.47 [0.30-0.74]	.001	0.52 [0.32-0.83]	.006
**Cytoreductive surgery at relapse**	No	226				
	Yes	294	0.49 [0.38-0.62]	<.001	0.63 [0.47-0.84]	.002
**TFI between the two last platinum-chemotherapy lines**	>12 months	421				
	≤12 months	99	2.83 [2.13-3.74]	<.001	2.68 [1.89-3.81]	<.001

Abbreviations: 95CI, 95% confidence interval; HR, hazard ratio; *N*, Number of patients; TFI, time-free interval.

## Discussion

We present here a large real-world cohort of patients with platinum-sensitive recurrent epithelial OvC receiving PARPi (olaparib, niraparib, and rucaparib gathered in a single population) as maintenance therapy after platinum rechallenge. The results provide valuable insights into the PFS (HR = 0.71; 95CI [0.55-0.93], *P = *0.01) and OS (HR = 0.47, 95CI [0.30-0.74], *P* < 0.001) outcomes, with survival improvement independent from other clinical and histological features such as performance status, achievement of cytoreductive surgery at relapse, and platinum-free interval duration before relapse.

Only few studies have been published in this setting.^20–22^ A recent Spanish expanded access program exploring niraparib efficacy in the recurrent setting included 316 patients with similar clinical features. These patients had a good performance status (all were PS 0 or 1), they had mostly *BRCA* wild-type tumors (80%), and 23% underwent debulking surgery at relapse. Rates of *BRCA*mut were higher in randomized clinical trials without *BRCA* mutation as inclusion criteria. For example, *BRCA*mut rate was 35% in the ARIEL-3 study exploring rucaparib efficacy in the same setting.^9^ Despite the cytoreductive surgery at relapse can be proposed in selected patients and has been shown to improve survival,^23,24^ surgical treatment at relapse was not described in pivotal studies exploring PARPi. It was achieved in 20%-32% of cases with data available in our set, similar to other real-world cohorts.^22^

The findings of this study confirm the clinical benefit of PARPi in the real-world setting, consistent with previous randomized controlled trials or observational cohorts. The median PFS was 19.9 months in patients receiving PARPi in our set (including 4 months of chemotherapy and a 2y-OS rate of 85%), significantly longer than the control group with a median PFS of 13.4 months and a 2y-OS rate of 74%. Considering the lack of selection based on *BRCA* status in the whole population, our results were close to other cohorts. Median PFS from PARPi initiation was 8.6 months in another unselected real-world cohort, 14 months in a retrospective cohort of *BRCA*mut patients, and 9.3 to 21.0 months in *BRCAwt* and *BRCAmu*t patients in the NOVA trial.^8,22,25^ However, the hazard ratio for PFS was 0.71 in our study, which is lower than what was observed in pivotal trials: HR = 0.45 in *BRCA*wt patients in the NOVA trial, and HR = 0.36 in the intention-to-treat population of the ARIEL-3 [8,9]. However, taking into account the retrospective nature of this study compared to the phase 3 prospective trials, these results confirm that PARPi improves PFS even in a real-world setting.

Our study demonstrates a significant improvement in OS with PARPi maintenance therapy. The median OS in the PARPi group was 82.0 months compared to 44.7 months in the control group. This is higher than the long-term OS results obtained in the SOLO-2 trial, with median OS with olaparib in *BRCA*mut tumors reaching 51.7 months, and 45.9 with rucaparib in a similar population.^10,13^ This discrepancy should be interpreted with caution because of limited follow-up in the PARPi group with a median follow-up of 24.7 months. However, patients included in our cohort seem to display more favorable features. More than 80% of the patients in our set had a TFI > 12 months whereas it was close to 60% in clinical trials,^9,10,12^ suggesting that they may display higher benefits to PARPi as platinum sensitivity may considered as a predictive marker of PARPi efficacy.^26^ Moreover, more than half of our population undergo cytoreductive surgery at relapse, which has been described to improve survival in this setting.^23^ Nevertheless, these results indicate that PARPi treatment may have a positive impact on long-term survival outcomes in patients with recurrent ovarian cancer based on prior platinum sensitivity. OS was indeed not significantly improved in pivotal trials: HR = 0.74 (not significant-ns), 0.85 (ns), and 0.83 (ns) with olaparib, niraparib, and rucaparib in *BRCA*mut patients; and HR = 1.06 (ns) with niraparib in *BRCA*wt cases or 0.99 (ns) with rucaparib in unselected patients.^10,12,13^ Another explanation for these inconsistent OS results may be differences in post-progression treatments. In randomized trials, 40%-50% of patients included in the placebo arm later received PARPi whereas none of the patients in our control group received PARPi during their whole disease history. However, it is important to note that these findings should be interpreted with caution due to the limited follow-up in the PARPi subset and the lack of *BRCA* status knowledge in 45% of patients in the control group.

One of the advantages of our study compared to other published retrospective of prospective real-world studies is its large sample size allowing multivariate analyses exploring PARPi value according to other usual clinicopathological features. Because of the imbalance observed between groups concerning *BRCA1/2* mutation status or achievement of complete cytoreductive surgery at relapse, the positive effect of PARPi observed in univariate analysis could be questionable. However, results of multivariate analyses show that PARPi improves PFS and OS independently of performance status, histological subtype, achievement of cytoreductive surgery at relapse, and treatment-free interval before relapse. These results have not been previously published in other real-world cohorts.

The strength of this study lies in its real-world design, which reflects the diverse patient population encountered in routine clinical practice. By using the ESME-OC database, a comprehensive and prospectively maintained database, the study captured extensive and reliable data on patient demographics, cancer characteristics, treatments, and outcomes. However, some limitations should be acknowledged. The retrospective nature of the study and potential selection biases may influence the results. Potential biases are related to the methodology (filtering process and data centralization) of maintenance of the ESME OC data platform itself and could be discussed: selection bias, misinformation or misclassification bias, confounding, and lack of safety data. This is especially true in safety data. Indeed, we did not have access to safety details regarding hematological and digestive toxicities, known to be frequent with PARPi.^27^ However, it is worth noting that no secondary leukemia or myelodysplastic syndrome has been reported, consistent with recent prospective data with an incidence of 0.73% (21 events out of 4533 patients).^28^ Additionally, the medium sample size of the PARPi group should be considered, and further validation in larger cohorts is warranted. Our choice to keep local assessments (with clinical, radiological, and biological data) for PFS evaluation could have led to mistakes in PFS events observations. However, this was done to be as close as possible to daily routine practice and to the information and treatments patients actually received. The positive OS results reinforce observations concerning PFS and the positive impact of PARPi on patients’ outcome. Finally, the lack of molecular data concerning *BRCA* status and homologous recombination deficiency (assays not routinely performed during the selection period) prevented us from deeply exploring the prognostic and predictive value of molecular alterations in the whole population.

However, we observed a differential effect of *BRCA* status in the subset of patients with data available. To the best of our knowledge, we present here the first ever-published real-world data of PARPi efficacy according to B*RCA*m status in platinum-sensitive recurrent OvC. However, as in all pivotal studies, the PFS gains were higher in patients with *BRCA*mut tumors, with HR for PFS of 0.59. This 40% reduction in the risk of progression was lower than that of prospective randomized trials: HR = 0.23-0.30 in *BRCA*m populations of NOVA, ARIEL-3, and SOLO-2 trials. As this analysis could only be exploratory due to the small sample size of patients with *BRCA*m tumors, these results need further validation in larger cohorts. Concerning *BRCA*wt patients, our results are not in line with that of randomized trials with no PFS benefit in this population. This may be explained by the small sample of this exploratory subgroup. The relatively good outcome of *BRCA*wt patients who did not receive PARPi (median PFS = 15.2 months, vs 3.8 months in non-gBRCA patients enrolled in the placebo group of the NOVA trial^8^) could also have decreased the power of these exploratory analysis and our capacity to identify an outcome improvement in *BRCA*wt patients.

In conclusion, this real-world study provides evidence confirming the efficacy of PARPi maintenance therapy after platinum-sensitive relapse in ovarian cancer patients. The results demonstrate improved PFS and OS outcomes in the PARPi group compared to observation without maintenance treatment. These results support the use of PARPi as maintenance therapy in clinical routine practice in patients who do not receive PARPi in the first-line setting.

## Supplementary Material

oyaf075_suppl_Supplementary_Figures_1-2

## Data Availability

All anonymized clinical data can be shared by the authors upon reasonable request. The Unicancer Network, sponsor of the ESME database, has a long history of academic data sharing for research purposes: • Researchers have to submit a request to the sponsor directly or through the corresponding author of the article. The request should be written in a predefined format of a short synopsis indicating the objective of the research, the methodology intended to be used, including the statistical analysis plan, and the variables within the database required for the research. • A scientific board will review and approve the requests on a case-by-case basis. Only encoded datasets will be used, which enables us to fulfill legal and ethical obligations to protect patients while at the same time utilizing patient data in progressing medical research to its full potential in the best interests of public health. A specific agreement between the sponsor and the researcher is requested for data transfer. This data transfer agreement details both parts responsibilities to ensure the required level of data integrity and legal and ethical obligations.
