# Supplementary material for: PARP inhibitors as maintenance therapy in ovarian cancer after platinum-sensitive recurrence: real-world experience from the Unicancer network
Source: Oncologist. 2025 May 11;30(5):oyaf075. doi: 10.1093/oncolo/oyaf075 (PMC12065941; doi:10.1093/oncolo/oyaf075)
Supplement: oyaf075_suppl_Supplementary_Figures_1-2 [file oyaf075_suppl_supplementary_figures_1-2.docx]

PARP inhibitors as maintenance therapy in ovarian cancer after platinum-sensitive recurrence: real-world experience from the Unicancer network – SUPPLEMENTARY DATA

Nicolas Rippstein et al.,

**Corresponding author**

Dr Renaud Sabatier, MD, PhD

Department of Medical Oncology, Institut Paoli-Calmettes, 232 Boulevard Sainte Marguerite, 13009 Marseille, France

E-mail: sabatierr@ipc.unicancer.fr.

***
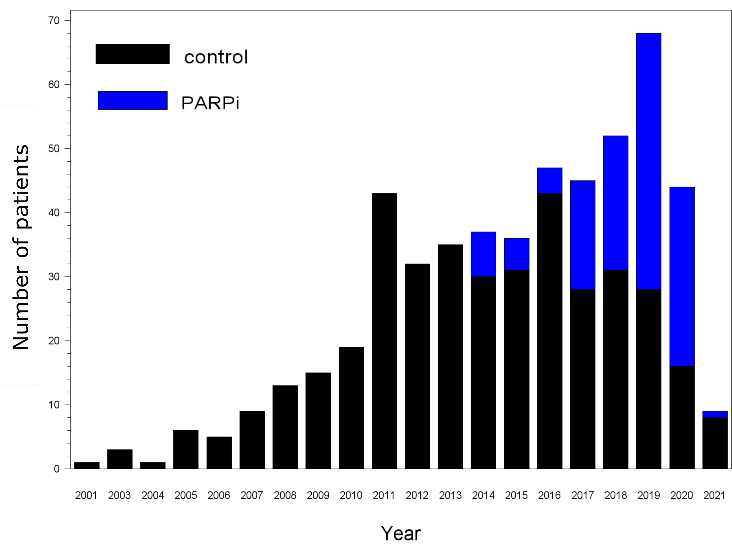
***

***Supplementary Figure 1.*** Histograms representing the number of inclusions in both groups by years. The inclusion date corresponds to the date of platinum chemotherapy prior to PARPi initiation in the PARPi group, and to the date of second-line platinum-chemotherapy in the control group. Some patients may have been included in the ESME database lately in their disease history but clinical data have been retrospectively added to the database.


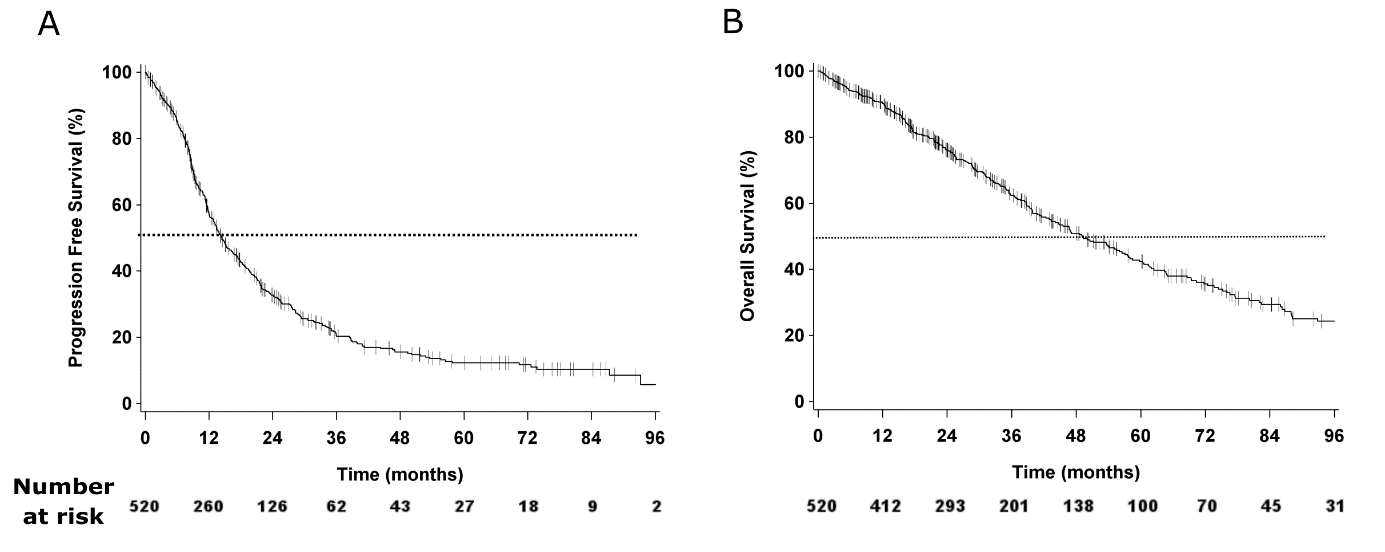


***Supplementary Figure 2.*** Kaplan Meier curves for Progression-free survival (A) and Overall Survival (B) in the whole population. The dashed horizontal lines indicate the median values.
